# *Burkholderia pseudomallei* Sequence Type 562 in China and Australia

**DOI:** 10.3201/eid2101.140156

**Published:** 2015-01

**Authors:** Hai Chen, Lianxu Xia, Xiong Zhu, Wei Li, Xiaoli Du, Duorong Wu, Rong Hai, Xiaona Shen, Ying Liang, Hong Cai, Xiao Zheng

**Affiliations:** Sanya People’s Hospital, Sanya, China (H. Chen, X. Zhu);; State Key Laboratory for Infectious Disease Prevention and Control, Beiiing, China (L. Xia, W. Li, , X. Du, R. Hai, X. Shen, Y. Liang, X. Zheng);; National Institute for Communicable Disease Control and Prevention, Beijing (L. Xia, W. Li, R. Hai, X. Du, X. Shen, Y. Liang, H. Cai, X. Zheng);; Collaborative Innovation Center for Diagnosis and Treatment of Infectious Diseases, Hangzhou, China ( L. Xia, W. Li, X. Zheng);; Haikou Municipal Hospital, Haikou, China (D. Wu)

**Keywords:** Melioidosis, *Burkholderia pseudomallei*, MLST, bacteria, China, Australia

**To the Editor:** Melioidosis is increasingly being recognized in tropical and subtropical areas worldwide; the world’s 2 major endemic foci are Thailand and northern Australia (*1*,*2*). Phylogenetic analyses of *Burkholderia pseudomallei* isolates, performed by using multilocus sequence typing (MLST) (*3*), have led to phylogeographic associations that can be used to track melioidosis epidemics (*4*). However, in contrast to the previous separation of *B. pseudomallei* into 2 phylogenetic groups (Australia and Southeast Asia/rest of the world) (*5*), we report an MLST sequence type (ST) that seems to be present in northern Australia, Taiwan, and southern China.

In mainland China, melioidosis was first reported in 1990 (*6*) and is now known to be endemic to several tropical provinces, including Hainan, a southern island province close to Southeast Asia. Since 2008, cases of melioidosis in Hainan have escalated; from July 2008 through July 2012, a total of 110 cases were microbiologically diagnosed at 2 general hospitals (Sanya People’s Hospital and Haikou Municipal Hospital). 

We characterized clinical isolates of *B. pseudomallei* from the 110 cases by using MLST, pulsed-field gel electrophoresis (PFGE), and 4-locus multilocus variable-number tandem-repeat analysis (MLVA-4) (*3*,*7*,*8*). MLST revealed 40 STs, 39 of which were consistent with STs from Southeast Asia, as evident from the global *B. pseudomallei* MLST database (http://bpseudomallei.mlst.net/). A single ST, ST562, which accounted for 3 cases in Hainan, was previously described on the global database as being from Australia; the 20 isolates from humans and 10 isolates from the environment deposited until September 1, 2014, all from Australia, had been isolated from 2005 through 2012. Although not deposited in the global MLST database, ST562 has also recently been reported from Taiwan (*7*). Among the 253 isolates of *B. pseudomallei* collected in Taiwan during 2004–2010, 1 clinical isolate and 9 environmental isolates were described as being ST562. Moreover, these 10 ST562 isolates displayed a unique PFGE pulsotype, distinct from that of other *B. pseudomallei* strains from Taiwan (*7*).

Of the 3 patients from Hainan from whom ST562 strains were isolated, 2 resided in the city of Sanya and 1 in the neighboring city of Lingshui (Technical Appendix); all denied a history of foreign travel, they shared no common risk factors, and all survived the infection. Further analysis of ST562, performed by using eBURST-based (http://eburst.mlst.net/) population analysis of the MLST dataset, showed that ST562 is a single-locus variant of ST167, which is represented on the MLST dataset by multiple human and environmental isolates from Thailand and to date by 1 human isolate from Cambodia. ST167 accounted for 1 of the 110 *B. pseudomallei* strains from Hainan. The *narK* locus of ST167contains allele 3 instead of allele 29, as seen in ST562; 3 base differences are found in allele 3: C72T (C→T position 72), C126T, and A435G. According to PFGE, the 3 ST562 isolates from Hainan displayed a single pulsotype, and the other 107 isolates from Hainan belonged to distinct and diverse pulsotypes, similar to those observed in Taiwan. The uniformity of PFGE patterns in the Hainan and Taiwan isolates supports the possibility that ST562 might be a recently emerging clone. PFGE patterns of Hainan ST562 exhibited 86% similarity with ST167, differing by 6 bands (Figure).

**Figure:**
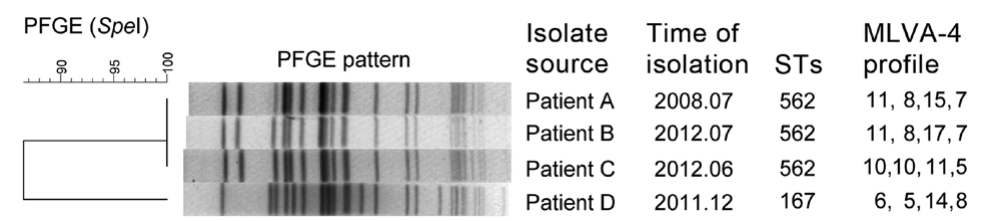
Pulsed-field gel electrophoresis (PFGE) patterns for 3 sequence type (ST) 562 and 1 ST167 *Burkholderia pseudomallei* strains isolated during 2008–2012, Hainan, China. The isolate source, isolation time, ST, and 4-locus multilocus variable-number tandem-repeat analysis (4-MLVA) profiles are indicated for each strain. Scale bar indicates percentage similarity.

Hainan ST562 isolates were further analyzed by using MLVA-4 (*8*), which divided 3 isolates (from patients A, B, and C) into 3 distinct MLVA-4 types (Figure). The 2008 isolate (MLVA-4 profile 11,8,15,7) and one 2012 isolate (profile 11,8,17,7) exhibited close relatedness, whereas another 2012 isolate (profile 10,10,11,5) was divergent from these, indicating that ST562 isolates in Hainan have been present long enough for some divergence into lineages.

Two mutually exclusive gene clusters, *B. thailandensis*–like flagellar gene cluster (BTFC) and *Yersinia*-like fimbrial gene cluster (YLF), have been linked to geographic origin and have been suggested for differentiating groups of *B. pseudomallei* (*9*). By PCR we found that ST562 isolates of Hainan were all YLF positive. BTFC predominates in Australian *B. pseudomallei* strains, and YLF predominates in Southeast Asia. Presence of YLF was also observed in strains from Papua New Guinea, possibly reflecting that country’s location, intermediate between major foci of melioidosis (*10*).

In conclusion, by using MLST and the online MLST database, we revealed that *B. pseudomallei* ST562 is present in southern China as well as in Australia and Taiwan. The intercontinental character of this ST raises new questions about the epidemiology and control of melioidosis. Given the usual geographic separation of *B. pseudomallei* STs, we suggest that this wide-ranging presence of ST562 might result from more recent spread caused by transmission between regions. Increasing farming exchanges and trade of agricultural products between melioidosis-endemic regions might facilitate breaking of the geographic barrier; clonal introduction of *B. pseudomallei* could potentially occur in new locations. Improved and cooperative surveillance is required for elucidating the current and future global dispersion range of *B. pseudomallei* and for monitoring the consequent melioidosis infections.

Technical Appendix Geographic distribution of 43 *Burkholderia pseudomallei* sequence type 562 strains identified in Australia; Taiwan; and Hainan, China, during 2004–2012.
