# Supplementary material for: Burkholderia pseudomallei Sequence Type 562 in China and Australia
Source: Emerg Infect Dis. 2015 Jan;21(1):166–8. doi: 10.3201/eid2101.140156 (PMC4285272; doi:10.3201/eid2101.140156)
Supplement: Technical Appendix — Geographic distribution of 43 Burkholderia pseudomallei sequence type 562 strains identified in Australia; Taiwan; and Hainan, China, during 2004–2012. [file 14-0156-Techapp-s1.pdf]

Article DOI: http://dx.doi.org/10.3201/eid2101.140156

Burkholderia pseudomallei Sequence Type 562 in China and Australia

Technical Appendix

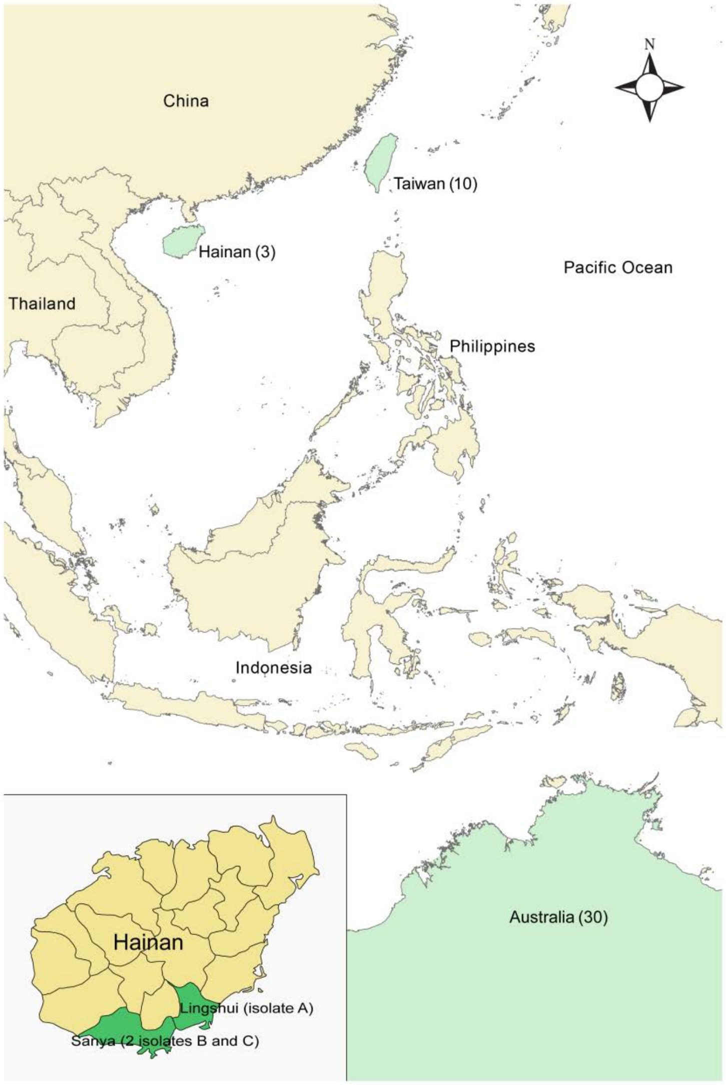

Appendix Figure. Geographic distribution of 43 *Burkholderia pseudomallei* sequence type 562 strains identified in Australia; Taiwan; and Hainan, China, during 2004–2012.Numbers in parentheses indicate the number of isolates from each region.
